# Association of Proteinuria Threshold in Pre-Eclampsia with Maternal and Perinatal Outcomes: A Nested Case Control Cohort of High Risk Women

**DOI:** 10.1371/journal.pone.0076083

**Published:** 2013-10-10

**Authors:** Kate Bramham, Carlos E. Poli-de-Figueiredo, Paul T. Seed, Annette L. Briley, Lucilla Poston, Andrew H. Shennan, Lucy C. Chappell

**Affiliations:** 1 Division of Women’s Health, Women’s Health Academic Centre, King’s College London and King’s Health Partners, London, United Kingdom; 2 School of Medicine, Pontificia Universidade Catolica do Rio Grande do Sul, Rio de Janeiro, Brazil; Indiana University School of Medicine, United States of America

## Abstract

**Objectives:**

To evaluate occurrence of adverse maternal and perinatal outcomes with different thresholds of proteinuria (300-499mg and ≥500mg/24 hours) in pre-eclamptic women, comparing outcomes against women with chronic and gestational hypertension.

**Design:**

Secondary analysis of the Vitamins in Pre-Eclampsia Trial.

**Setting:**

25 UK hospitals in ten geographical areas.

**Population:**

946 women with pre-existing risk factors for pre-eclampsia.

**Methods:**

Women with pre-eclampsia and proteinuria 300-499mg/24h (PE300, referent group, n=60) or proteinuria ≥500 mg/24h (PE500, n=161) were compared with two groups of non-proteinuric women with chronic hypertension (CHT, n=615) or gestational hypertension (GH, n=110).

**Main Outcome Measures:**

Maternal: progression to severe hypertension. Perinatal: small for gestational age (SGA) <5^th^ centile, gestation at delivery.

**Results:**

Severe hypertension occurred more frequently in PE500 (35%) and PE300 (27%) than CHT (5.9%; P≤0.01) and GH (10%; p≤0.001). Gestation at delivery was earlier in PE500 (33.2w) than PE300 (37.3w; P≤0.001), and later in CHT (38.3w; P≤0.05) and GH (39.1w; P≤0.001). SGA infants were more frequent in PE300 (32%) than in CHT (13.3%; P≤0.001) and GH (16.5%; P≤0.05). Women in PE500 were more likely to have a caesarean section than PE300 (78% vs. 48%; P≤0.001), and to receive magnesium sulphate (17% vs. 1.7%, P≤0.05).

**Conclusion:**

Women with PE300 have complication rates above those of women managed as out-patients (GH and CHT), meriting closer surveillance and confirming 300 mg/d as an appropriate threshold for determining in-patient management. Adverse perinatal outcomes are higher still in women with PE500.

## Introduction

Proteinuria is a criterion for diagnosis in the most widely accepted definitions of pre-eclampsia, but the threshold for significance remains controversial [1,2]. Recent guidelines from the National Institute for Health and Clinical Excellence (NICE) for the management of hypertensive disorders in pregnancy have highlighted that the prognostic value of different quantities of urinary protein is unclear and recommend research to identify diagnostic thresholds of proteinuria which are accurate in predicting clinically important outcomes, thus allowing interventions based on these thresholds to be evaluated in randomised controlled trials [3] .

Women with gestational hypertension and ‘significant’ proteinuria are commonly admitted for observation until delivery, which has implications for clinical management and health care resources[4]. International guidelines support the upper limit of normal proteinuria during pregnancy as 300 mg/24h [5], but evidence for an association with adverse outcomes is not strong [6]. Some authors including those of the Canadian Hypertension Committee Guidelines for management of hypertensive disorders of pregnancy [7], consider a threshold of 300mg/ 24h to be a poor predictor of clinical outcome and that 500 mg/24h, or a protein/creatinine ratio of 0.5 mg/mg, is a better discriminator of women at risk of adverse outcomes [8,9]. Furthermore because of the uncertainty that the degree of proteinuria is outcome-related, the NICE guidelines suggest that repeated quantification of proteinuria is not necessary other than to make an initial diagnosis of proteinuria [3].

We have therefore undertaken a study, using a nested case-control design of women who participated in the Vitamins In Pre-eclampsia (VIP) trial[10], to determine which proteinuria threshold is important for clinical management in pre-eclampsia in high-risk women. The specific objective was to evaluate the occurrence of adverse maternal and perinatal outcomes with different thresholds of proteinuria (300-499 and ≥500 mg/24h) in women with pre-eclampsia. The clinically relevant comparator group was chosen as high-risk women currently managed in an outpatient setting (those with pre-existing chronic hypertension without additional proteinuria and those with gestational hypertension) in order to assess whether women with 300-499 mg/ 24h of proteinuria could be considered comparable and therefore suitable for outpatient management. 

## Methods

The VIP trial was a randomized placebo controlled trial of vitamin C and E supplementation to prevent pre-eclampsia in women at increased risk [10]. 2410 women from 25 hospitals were enrolled between 6 August 2003 and 27 June 2005. The South East Multi Ethics Research Committee provided ethics approval (number 00/01/027), and site specific approval for each participating centre was given. Written informed consent was obtained from all participants. 

The VIP trial inclusion criteria were gestational age 14^+0^ to 21^+6^ weeks together with one or more of the following risk factors: pre-eclampsia in the pregnancy preceding the index pregnancy requiring delivery before 37 completed weeks’ gestation, diagnosis of HELLP syndrome (haemolysis, elevated liver enzymes, and low platelets) or eclampsia in any previous pregnancy at any stage of gestation; essential hypertension requiring medication, currently or previously; maternal diastolic blood pressure of 90 mm Hg or more before 20 weeks’ gestation in the current pregnancy; type 1 or type 2 diabetes, requiring insulin or oral hypoglycaemic therapy before the pregnancy; antiphospholipid syndrome; chronic renal disease (creatinine ≥125 μmol/L pre-pregnancy or ≥100 μmol/L during pregnancy, or significant proteinuria (≥500 mg per 24 h)); multiple pregnancy; abnormal uterine artery Doppler waveform (18–22 weeks’ gestation, mean resistance index >0·67 or pulsatility index >1·65 with or without the presence of unilateral or bilateral diastolic notches); primiparity with body-mass index (BMI) at first antenatal appointment of 30 kg/m^²^ or more. Women unable or unwilling to give written informed consent, being treated with warfarin or taking vitamin supplements with more than 200 mg of vitamin C or more than 40 IU of vitamin E daily were excluded. 

For the purposes of this analysis, women with twin pregnancies, pre-existing proteinuria and those women with pre-eclampsia without quantification of proteinuria by 24-hour urine collection and women who developed HELLP syndrome were excluded.

Definitions: Gestational hypertension: two or more readings of a diastolic blood pressure of 90 mm Hg or more taken at least 4 h and up to 168 h apart and occurring after 20 weeks of pregnancy, excluding labour; chronic hypertension: on antihypertensive treatment pre-pregnancy or as defined above, but occurring before 20 weeks’ gestation; severe hypertension: diastolic blood pressure >110mmHg; pre-eclampsia: gestational hypertension with proteinuria; proteinuria: excretion of 300 mg protein or more over 24 h; chronic hypertension with superimposed pre-eclampsia: chronic hypertension with new development of proteinuria; severe pre-eclampsia: severe gestational hypertension with proteinuria; HELLP: haemolysis, elevated liver enzymes and low platelets syndrome and eclampsia (seizures) also meeting pre-eclampsia criteria. Proteinuria was quantified when 1+ or more was detected on routine dipstick urinalysis. 

Women were allocated into one of four groups: pre-eclampsia with maximal quantified proteinuria of 300-499 g/24h (PE300, n=60), pre-eclampsia with proteinuria of at least 500 mg/24h (PE500, n=161), non-proteinuric chronic hypertension without pre-eclampsia (CHT, n=615), non-proteinuric gestational hypertension (GH, n=110). Women with chronic hypertension who developed superimposed pre-eclampsia were included in the appropriate pre-eclampsia group according to maximal level of proteinuria.

The following maternal outcomes were evaluated by detailed case note review: gestational age, severe gestational hypertension, labour onset (spontaneous, induction, pre-labour caesarean section), delivery mode (spontaneous vaginal, instrumental vaginal, caesarean section), use of magnesium sulphate, use of anti-hypertensive therapy (any treatment and intravenous therapy), antenatal steroids, intensive care unit admission, steroid for maternal reasons and number of courses. The occurrence of eclampsia, HELLP, maternal deaths, stroke, cerebral haemorrhage, liver rupture, pulmonary edema, and disseminated intravascular coagulation was also determined. For complex cases, the trial management team reviewed the case and a diagnosis was confirmed by two senior clinical staff, acting independently.

Neonatal outcomes included perinatal deaths, pre-term birth (<37 and 34 weeks gestation, spontaneous or iatrogenic), use of surfactant, mechanical ventilation and respiratory distress. Birth weight was assessed by customized birth weight percentile (gestation related Optimal Weight) [11], and small for gestational age (SGA) reported as <10^th^ and <5^th^ centiles.

Highest antenatal serum aspartate aminotransferase (AST) and creatinine, and lowest antenatal platelet count were recorded; women had haematological and biochemical testing performed for clinical indications.

Data are presented as means (standard deviation) or frequency (percentages), and medians (interquartile ranges) for laboratory parameters which were not normally distributed. Comparisons of outcomes between groups are presented as differences in means (95% confidence intervals) for continuous measures and as risk ratios (95% confidence intervals) for percentages. Risk ratios are presented if there were more than ten women in each of the four groups for comparison. Geometric means of laboratory parameters between groups were compared with Mann Whitney tests. Bootstrapping was used to develop confidence intervals for the difference in the arithmetic means for indices of healthcare resources (maternal and neonatal inpatient stay)[12]. The group PE300 was considered the reference group for comparisons. Risk ratios were adjusted for age and ethnicity if there were no missing values. For outcomes with significant differences between groups, odds ratios were calculated with adjustment for the presence of severe hypertension, highest antenatal creatinine, and lowest antenatal platelets. A probability of ≤0.05 was considered to be statistically significant. Stata software (version 10.1; StataCorp, College Station, Texas, USA) was used for statistical analysis.

The study is reported according to STROBE guidelines. 

## Details of Ethics Approval

The South East Multi Ethics Research Committee provided ethics approval (number 00/01/027), and site specific approval for each participating centre was given. Written informed consent was obtained from all participants. 

## Results

A total of 948 women with singleton pregnancies were identified; one woman was excluded due to incomplete outcome data. Baseline demographics, risk factors and medications at entry are shown in Table 1. Women with CHT were less likely to have had previous pre-eclampsia/HELLP or eclampsia than women with PE300 or PE500 (i.e. recurrent pre-eclampsia) or GH (P<0.0001). Black ethnicity was more common in both PE300 and PE500 than CH or GH groups (P≤0.001).

**Table 1:** Demographics, risk factors and medications at enrolment to study.

	Preeclampsia	Preeclampsia	Chronic Hypertension	Gestational Hypertension
	300-499 mg/day	≥500 mg/day		
N	60	161	615	110
Gestational age at recruitment, weeks	18.7 (2.4)	18.1 (2.5)	18.4 (2.5)	18.4 (2.5)
Maternal age, years	32.5 (5.5)	31.6 (5.6)	33.0 (5.0)	29.6 (5.6)
Mother's ethnic group (ONS categories), N (%)				
Asian	4 (6.6%)	12 (7.4%)	26 (4.2%)	2 (1.8%)
Black	15 (24.6%)*	35 (21.5%)*	72 (11.7%)*	5 (4.5%)*
White	37 (60.7%)	110 (68.3%)	506 (82.3%)	99 (90.0%)
Other	4 (6.7%)	4 (2.5%)	11 (1.8%)	4 (3.6%)
Smoking status, N (%)				
Current smoker>=1/day	5 (8.2%)	13 (8.0%)	44 (7.2%)	14 (12.7%)
Stopped during pregnancy	5 (8.2%)	19 (11.7%)	40 (6.5%)	9 (8.2%)
Weight, Kg	84.9 (20.3)	82.8 (20.5)	82.5 (20.7)	88.0 (20.5)
BMI, Kg/m^2^	31.7 (6.8)	30.7 (7.4)	30.5 (7.1)	32.8 (7.3)
BMI categories, N (%)				
<25 Kg/m^2^	13 (21.3%)	38 (23.3%)	155 (25.2%)	19 (17.3%)
25<30 Kg/m^2^	16 (26.2%)	51 (31.3%)	166 (27.0%)	12 (10.9%)
30-35 Kg/m^2^	11 (18.0%)	33 (20.2%)	143 (23.3%)	40 (36.4%)
>35 Kg/m^2^	21 (34.4%)	41 (25.2%)	151 (24.6%)	39 (35.5%)
Proteinuria/ 24 hours (mg) Median (IQR)	382 (364-427)	1300 (1100-1440)	-	-
Systolic Blood Pressure at baseline, mmHg	127.2 (14.8)	130.8 (13.5)	131.1 (14.8)	123.8 (12.4)
Diastolic Blood Pressure at baseline, mmHg	79.4 (10.4)	80.1 (11.1)	81.4 (10.5)	75.3 (7.2)
Co-existing condition in pregnancy				
Diabetes	2 (3.3%)	20 (12.3%)	27 (4.4%)	6 (5.5%)
Previous HELLP/Eclampsia/Preeclampsia	23 (37.7%)**	769 (42.9%)**	119 (19.3%)**	44 (40.0%)**
Systemic Lupus Erythematosus	0 (0%)	2 (1.2%)	7 (1.1%)	2 (1.8%)
Abnormal Uterine artery Doppler	6 (9.8%)	8 (4.9%)	15 (2.4%)	5 (4.5%)
Medications at entry				
Aspirin, N (%)	22 (36.7%)	56 (34.8%)	210 (34.1%)	24 (21.8%)
Heparin, N (%)	3 (4.9%)	3 (1.8%)	19 (3.1%)	3 (2.7%)
Antihypertensive therapy at booking, N (%)	19 (31.7%)	48 (29.8%)	239 (38.9%)	0 (0.0%)
Antihypertensive therapy prior to pregnancy, N (%)	19 (31.7%)	54 (33.5%)	321 (52.2%)	0 (0.0%)

Data are given as mean (standard deviation) or number (percentage). Abbreviations: ONS: Office for National Statistics; BMI: Body Mass Index;

* PE300 and PE500 compared with both CH or GH groups P<0.0001; ** CHT compared with PE300, PE500 and GH P<0.0001

Table 2 presents maternal outcomes using the PE300 group as reference. Severe hypertension was more frequent in PE300 and PE500 than in CHT and GH groups (CHT P≤0.001, GH P≤0.01). Women with PE500 were more likely to receive magnesium sulphate than women with PE300 (PE300 P≤0.05). Women with PE300 were more likely to have higher ALT than women with CHT P≤0.05) and lower platelet counts that women in the CHT and GH groups (CHT P≤0.05, GH P≤0.01). 

**Table 2:** Principal maternal outcomes by level of proteinuria adjusted for age and ethnicity with risk ratios in comparison with PE 300 group (proteinuria 300- 499 mg/24 hours).

	Preeclampsia	Preeclampsia	Chronic Hypertension	Gestational Hypertension
	300-499 mg/day	≥500 mg/day		
**N**	60	161	615	110
Gestational age at diagnosis of preeclampsia, weeks	35.2 (3.8)	33.2 (4.1)	-	-
Severe gestational hypertension, N (%)	16 (26.7%)	58 (34.8%)	36 (5.9%)	11 (10.0%)
Diastolic blood pressure >110mmHg Risk ratio		1.30 (0.82 to 2.09)	0.22 (0.13 to 0.37)***	0.38 (0.19 to 0.76)**
Maternal Intensive care unit admission, N (%)	1 (1.7%)	4 (2.5%)	3 (0.5%)	1 (0.9%)
**Medication use after enrollment, N (%)**				
Parenteral anti-hypertensive use	2 (3.3%)	13 (8.1%)	8 (1.3%)	3 (2.7%)
Magnesium sulfate use	1 (1.7%)	28 (17.4%)	5 (0.8%)	1 (0.9%)
Risk Ratio		10.43 (1.45 to 75.01) *		
Antenatal steroids	17 (28.3%)	64 (39.8%)	41 (6.7%)	6 (5.5%)
			0.24 (0.14 to 0.39)***	0.19 (0.08 to 0.46)***
**Laboratory Investigations**				
**N**	52	149	369	80
Highest antenatal alanine aminotransferase (IU/L) (ALT) (Median, IQR)	17(13.25 to 29.75)	17 (11.5 to 26.5)	16 (12 to 22) ^*^	15 (11 to 22)
Risk Ratio			0.78 (0.63 to 0.96)	
ALT >40IU/L , N(%)	8 (15.4%)	18 (12.1%)	24 (6.5%)	8 (10.0%)
**N**	59	1160	452	99
Lowest antenatal platelets (x10^9^/L) (Median, IQR)	210 (171 to 276)	224.5 (176 to 264)	241 (204 to 286)*	254 (218 to 284) ^**^
Risk Ratio			1.10 (1.02 to 1.20)	1.15 (1.05 to 1.26)
Platelets <100 x10^9^/L N(%)	1 (1.7%)	3 (1.9%)	3 (0.7%)	0
**N**	55	161	386	93
Highest antenatal Creatinine (µmol/L) (Median, IQR)	69 (62 to 77)	75 (66 to 87) ^**^	67 (60 to 74)	68 (61 to 75)
Risk Ratio		1.11 (1.04 to 1.19)		
Creatinine >90µmol/l, N(%)	4 (7.1%)	30 (18.4%)	13 (3.4%)	7 (7.5%)

Data are given as mean (standard deviation) or number (percentage) together with risk ratios and 95% confidence intervals (CI).

Risk ratio, p in comparison with preeclampsia with proteinuria of 300 - 499 mg/24 hours; *P≤0.05; **P≤0.01; ***P≤0.001

Delivery outcomes are presented in Table 3. Women with PE500 were delivered almost two weeks earlier than the PE300 group (P≤0.001). Women with PE500 were more likely to have a caesarean section (P≤0.001), including pre-labour caesarean section (P≤0.01) and less likely to be induced than those in the PE300 group (P≤0.001). Women with chronic hypertension (CHT) were delivered approximately one week later (P≤0.05) than the PE300 group and those with gestational hypertension two weeks later (P≤0.001) . Both women with CHT and GH were less likely to be induced than women in the PE300 group (CHT P≤0.001, GH P≤0.05). Women in the PE500 had a higher risk of caesarean delivery than women with CHT and GH (CHT (P≤0.001), GH (P≤0.001), pre-labour caesarean section (CHT P≤0.001, GH (P≤0.001)).

**Table 3:** Principal delivery outcomes by level of proteinuria adjusted for age and ethnicity with risk ratios in comparison with PE 300 group (proteinuria 300- 499 mg/24 hours).

	Preeclampsia	Preeclampsia	Chronic Hypertension	Gestational Hypertension
	300-499 mg/day	≥500 mg/day		
**N**	60	161	615	110
**Labour onset, N (%)**				
Spontaneous	5 (8.3%)	8 (5.0%)	240 (39.0%)***	32 (29.1%)**
Risk ratio			4.68 (2.01 to 10.90)	3.49 (1.44 to 8.49)
Induction	37( 61.7%)	60 (37.3%)	214 (34.8%)	48 (43.6%)
Risk ratio		0.60 (0.46 to 0.81)***	0.56 (0.45 to 0.71)***	0.71 (0.53 to 0.95)*
Pre-labour caesarean section	18 (30.0%)	93 (57.8%)	161 (26.2%)	30 (27.3%)
Risk ratio		1.93 (1.28 to 2.90)**	0.87 (0.58 to 1.31)	0.91 (0.56 to 1.49)
**Delivery mode, N (%)**				
Spontaneous vaginal delivery	23 (38.3%)	27 (16.8%)	282 (45.9%)	46 (41.8%)
Instrumental vaginal delivery	8 (13.3%)	8 (5.0%)*	64 (10.4%)	12 (10.9%)
Risk Ratio		0.37 (0.15 to 0.95)		
Caesarean section	29 (48.3%)	126 (78.3%)	269 (43.7%)	52 (47.3%)
Risk ratio		1.62 (1.23 to 2.13) ***	0.90 (0.69 to 1.19)	0.98 (0.70 to 1.36)
Gestational age at delivery, weeks	37.3 (3.2)	33.2 (4.1)	38.3 (3.7)	39.1 (1.7)
Mean difference (95% CI)		- 2.02 (-3.01 to -1.03) ***	0.99 (-0.13 to 1.84) *	1.77 (0.89 to 2.64) ***

Data are given as mean (standard deviation) or number (percentage) together with risk ratios and 95% confidence intervals (CI).

Risk ratio: comparison with preeclampsia with proteinuria of 300–499 mg/24 hours; *P≤0.05; **P≤0.01; ***P≤0.001

There were two maternal deaths (PE300 and CHT groups: a suicide following an episode of acute psychosis at day 5 postpartum and a death at 3 months postpartum in an HIV positive woman which was unrelated to her CHT), one case of eclampsia (PE500), two cases of pulmonary edema (PE500 and CHT) and one case of disseminated intravascular coagulation (CHT).

Neonatal outcomes are described in Table 4. Women in the PE500 group were more likely to have preterm deliveries (P≤0.001) (including iatrogenic preterm delivery (P≤0.001) ) or to have an infant <5^th^ centile (P≤0.05) than women in the PE300 group, but other perinatal outcomes were not significantly different. Compared to women in the PE300 group, women with chronic hypertension were less likely to deliver preterm (P≤0.05), to have an SGA infant (P≤0.001) and women with gestational hypertension were also less likely to have an SGA infant (P≤0.01). There was no difference in the proportion of perinatal deaths between the groups. 

**Table 4:** Principal perinatal outcomes by level of proteinuria adjusted for age and ethnicity with risk ratios in comparison with PE 300 group (proteinuria 300- 499 mg/24 hours).

	Preeclampsia	Preeclampsia	Chronic Hypertension	Gestational Hypertension
	300-499 mg/day	≥500 mg/day		
N	60	161	615	110
SGA (<10th adjusted centile), N (%)	24 (40.0%)	84 (52.5%)	122 (20.1%)	28 (25.7%)
Risk ratio		1.30 (0.93 to 1.82)	0.49 (0.35 to 0.69)***	0.63 (0.40 to 0.97)*
SGA (<5th adjusted centile), N (%)	19 (31.7%)	79 (49.4%)	81 (13.3%)	18 (16.5%)
Risk ratio		1.56 (1.04 to 2.33)*	0.48 (0.32 to 0.72)***	0.48 (0.32 to 0.72)*
Total deaths, N (%)	2 (3.3%)	4 (2.5%)	21 (3.4%)	0 (0.0%)
Preterm delivery (<37w), N (%)	15 (25.0%)	99 (61.5 %)	83 (13.5%)	10 (9.1%)
Risk ratio		2.41 (1.56 to 3.89)***	0.60 (0.38 to 0.95)*	0.51 (0.23 to1.10)
Spontaneous preterm delivery, N (%)	2 (3.3%)	4 (2.5%)	32 (5.2%)	4 (3.6%)
Iatrogenic preterm delivery, N (%)	13 (21.7%)	95 (59.0%)	51 (8.3%)	6 (5.5%)
Risk ratio		2.72 (1.66 to 4.48)***	0.41 (0.24 to 0.71)***	
Preterm delivery < 34 weeks, N (%)	8 (13.3%)	43 (26.7%)	41 (6.7%)	0 (0.0%)
Risk ratio		1.97 (1.01 to 3.85)*	0.64 (0.32 to 1.28)	
Surfactant use, N (%)	2 (3.3%)	21 (13.0%)	15 (2.4%)	0 (0.0%)
Mechanical Ventilation, N (%)	4 (6.7%)	20 (12.4%)	15 (2.4%)	1 (0.9%)
Respiratory distress syndrome, N (%)	5 (8.3%)	30 (18.6%)	18 (2.9%)	0 (0.0%)

Risk ratio: comparison with preeclampsia with proteinuria of 300–499 mg/24 hours; *P≤0.05; **P≤0.01; ***P≤0.001

Even with adjustment for the effects of severe hypertension, creatinine and platelets, women with PE500 still were significantly more likely than PE300 to have preterm delivery <37 weeks (P≤0.001). Women with PE300 were also more likely to have SGA <5th (P≤0.01) and 10th centile infants (P≤0.01), and preterm deliveries <34 weeks (P≤0.05) than women with CHT, and more likely to have preterm deliveries <37 weeks than women with GH (P≤0.01) . Odds ratios are presented in Table 5.

**Table 5:** Principal perinatal outcomes by level of proteinuria adjusted for presence of severe hypertension (Diastolic blood pressure >110mmHg), platelet count, alanine aminotransferase and creatinine with odds ratios in comparison with PE 300 group (proteinuria 300- 499 mg/24 hours).

	Preeclampsia	Preeclampsia	Chronic Hypertension	Gestational Hypertension
	300-499 mg/day	≥500 mg/day		
N	60	161	615	110
SGA (<10th adjusted centile), N (%)	24 (40.0%)	84 (52.5%)	122 (20.1%)	28 (25.7%)
Odds Ratio		1.44 (0.74 to 2.80)	0.40 (0.21 to 0.75)**	0.44 (0.20 to 0.97)*
SGA (<5th adjusted centile), N (%)	19 (31.7%)	79 (49.4%)	81 (13.3%)	18 (16.5%)
Odds Ratio		1.82 (0.92 to 3.61)	0.41 (0.21 to 0.79) **	0.42 (0.18 to 0.99) *
Preterm delivery (<37w), N (%)	15 (25.0%)	99 (61.5 %)	83 (13.5%)	10 (9.1%)
Odds Ratio		4.43 (2.14 to 9.20) ***	0.52 (0.25 to 1.06) *	0.21 (0.07 to 0.63) **
Preterm delivery < 34 weeks, N (%)	8 (13.3%)	43 (26.7%)	41 (6.7%)	0 (0.0%)
Odds Ratio		2.428 (0.97 to 6.07)	0.49 (0.19 to 1.27)	

Odds ratio: comparison with preeclampsia with proteinuria of 300–499 mg/24 hours; *P≤0.05; **P≤0.01; ***P≤0.001

## Discussion

The optimal place of management (inpatient vs. outpatient) has been debated for hypertensive women with low levels of proteinuria (5); this study has demonstrated that pre-eclamptic women with proteinuria 300-499 mg/24h and underlying risk factors for pre-eclampsia, cannot be considered to be at a risk level equivalent to women with chronic or gestational hypertension and these data do not support this group being managed as out-patients. Women with proteinuria above 500 mg/24h have worse pregnancy outcomes than those with 300mg/24h, including delivery nearly two weeks earlier, higher rates of caesarean section, pre-labour caesarean section, and requirement for magnesium sulphate, implying that proteinuria may be a surrogate marker for more severe disease. Current practice in the UK at the time of the study was to deliver a pre-eclamptic woman if she had uncontrollable blood pressure (on maximal triple therapy), had deteriorating haematological or biochemical parameters or if there was evidence of substantial fetal compromise (on fetal Doppler or cardiotocography). Women were not routinely delivered after a set time period from diagnosis and therefore the variance in gestational age at delivery between the groups is likely to represent differences in clinical condition rather than predetermined management strategy. The threshold of proteinuria above 300 mg/24h is associated with an increased likelihood of severe hypertension and SGA <5^th^ centile when compared with women with hypertension (chronic or gestational) but no proteinuria; these are outcomes related to the underlying disease rather than being driven by physician behaviour. Women are also more likely to be delivered earlier, due to iatrogenic intervention. This may represent clinical need (due to worsening hypertension and suspected fetal growth restriction) and/ or the use of guidelines recommending delivery when term gestation is reached. 

The large number of prospectively studied women is one of the major strengths of the study. Outcomes were collected by detailed case note review by dedicated researchers, rather than by relying on hospital coding which can be inaccurate. Only women at high risk of developing pre-eclampsia were included in the trial and therefore a limitation of the study is the lack of healthy controls. However, comparison between women with pre-eclampsia and women with hypertension (chronic or gestational), usually managed as outpatients, provides a more clinically useful comparison. Care was taken to exclude patients with renal disease, since they have higher risk of pregnancy related complications [13]. 

By some definitions, proteinuria is not required for the diagnosis of pre-eclampsia[14], and in others different thresholds for proteinuria to diagnose pre-eclampsia are given [5] [7,15-17]. Variable criteria and thresholds have been used in clinical guidelines and trials. The predominant threshold is either >300 mg/24h, PCR >30 mg/mmol or at least + to ++ on dipstick. The origin of the 300 mg/24h threshold of normal proteinuria in pregnancy is not clear; in one study the upper 95% confidence limit of the urinary protein concentration in 270 pregnant women was 260 mg/24h [18], which could infer any higher value is ‘abnormal’. A threshold of 500 mg/24h, or PCR of 0.5 mg/mg, has been suggested as a reference for significant proteinuria in pre-eclampsia [2,9,19], as a relevant value for intervention and hospitalizations [20], and a possible better predictor of outcome[7]. Whilst we recognise that other parameters including severity of hypertension, biochemical abnormalities and fetal compromise are likely to guide decisions regarding delivery than proteinuria, a clinical threshold to guide professionals in antenatal care is important, in order to facilitate admission women at higher risk of adverse events. For this reason we chose to evaluate thresholds used in current clinical practice, rather treating proteinuria as a continuous measure. Our results indicate that 300 mg/24h represents a clinically relevant threshold associated with greater pregnancy complications than in women with hypertension alone. Furthermore, the observation that proteinuria ≥500 mg/24h is associated with a higher risk of adverse events, provides evidence against its use as a threshold for diagnosis of pre-eclampsia, as this could result in high risk women being managed in an outpatient setting with detrimental consequences.

Thornton and colleagues compared maternal and fetal outcomes in 617 singleton pregnancies with pre-eclampsia diagnosed according to the Australasian Society of Study of Hypertension in Pregnancy Consensus Statement, and 417 were considered to have non-proteinuric pre-eclampsia. The proteinuric patients had earlier deliveries, were more likely to deliver by Caesarean section, and received more magnesium sulfate, which concurs with in our observations in women with proteinuria >500 mg/24h [21]. Also, in common with the present study, Chan and colleagues showed parallels between adverse maternal and fetal outcomes and higher levels of proteinuria in women with proteinuric pre-eclampsia, although a specific cut-off for use as a screening test was not identifiable [22]. 

A systematic review of selected studies with a proteinuria threshold >5g/24 hours suggested that proteinuria should not be used for clinical decisions [23]. Although the review concluded that this high level of proteinuria was unrelated to outcome, our study supports the concept that pre-eclamptic women with levels of proteinuria >500mg/24h are more likely to have operative deliveries and therapeutic interventions to avoid further complications. Our data suggest that the presence of proteinuria in women with hypertensive disorders of pregnancies is important and that the quantity of proteinuria is relevant to both maternal and neonatal outcomes, however there may be a threshold of proteinuria above which clinical outcomes are unchanged. 

Recently, the Pre-eclampsia Integrated Estimate of Risk Study (PIERS) study has evaluated the relationship between pregnancy outcome in pre-eclampsia and proteinuria in 2002 women, as assessed using dipsticks, spot urine protein: creatinine ratios or 24-hour urine collections[17]. In contrast to the present study, it was concluded that proteinuria was overall unrelated to outcome. Possible differences are that in the PIERS study pre-eclampsia was considered in the absence of proteinuria, if hypertension and hyperuricemia or HELLP syndrome were present. Indeed it is likely that the heterogeneity amongst these and many previous studies is a facet of the different diagnostic criteria employed in the definition of pre-eclampsia, which originates from incomplete comprehension of its pathophysiology and likely diverse aetiology[24]. Nevertheless in the PIERS study an increased odds of adverse outcomes was observed when 3 to 4+ dipstick proteinuria was detected [17]. 

An important distinction between our findings and others, are the additional co-morbidities in the women studied. It was not possible to adjust for all underlying risk factors, nor did we consider those without adverse pregnancy events to be a useful comparator given their diversity; however a higher proportion of women with PE500 had diabetes than women with PE300, reflecting a predisposition to proteinuria due to glomerular damage, although numbers are small. There was a higher proportion of black women in both PE groups than in GH and CHT groups. Similarly black ethnicity has also been reported to be associated with a higher prevalence of proteinuria in non-pregnant individuals [25] as well as adverse pregnancy outcomes [26,27]. However, despite adjustment for ethnicity, adverse outcomes remained significantly higher in PE300 and PE500 groups, suggesting high proteinuria is an important predictor of pregnancy outcome in those at risk. 

It is possible that some women in PE300 may have developed gestational proteinuria with pre-existing hypertension, rather than a true ‘pre-eclampsia’ syndrome, and therefore had more benign outcomes than the women in PE500. Confirming the diagnosis in such women is always challenging as there is no definitive biomarker present in pre-eclampsia. Chronic hypertension, and other risk factors such as obesity and previous pre-eclampsia, are associated with secondary focal segmental glomerulosclerosis [28-30]. Some women with pre-existing glomerular damage may have blood pressure and proteinuria in the upper ‘normal range’ that is exacerbated by physiological changes towards term, resulting in hypertension and proteinuria greater than diagnostic thresholds for pre-eclampsia, without placental disease, or systemic manifestations of the condition. 

Finally, it is important to recognize that the additional predisposing risk factors affecting the women studied may play a significant role in the overall pregnancy outcomes reported. Changing demographics, including increased rates of obesity, chronic hypertension and diabetes, all of which are associated with proteinuria as a marker of severity in the non-pregnant state, may ultimately alter the relationship between gestational proteinuria and pregnancy outcomes, and assessment of women for pre-eclampsia should be made in accordance with their underlying risk.

## Conclusion

Current practice is for proteinuria to be routinely assessed in women with hypertension in pregnancy. Our findings suggest that pre-eclamptic women with additional predisposing risk factors and proteinuria of 300-499mg/24h have more severe hypertension, early deliveries and SGA infants above those of women managed as out-patients (GH and CHT); those with proteinuria ≥500mg/24h should be considered to be at substantially greater risk of pregnancy complications than women with proteinuria of 300 mg/24h. The use of the 300mg/24h threshold for proteinuria in clinical practice as guidance for admission appears appropriate and should be continued.
